# LooplessFluxSampler: an efficient toolbox for sampling the loopless flux solution space of metabolic models

**DOI:** 10.1186/s12859-023-05616-2

**Published:** 2024-01-02

**Authors:** Pedro A. Saa, Sebastian Zapararte, Christopher C. Drovandi, Lars K. Nielsen

**Affiliations:** 1 Department of Chemical and Bioprocess Engineering, School of Engineering, Pontifical Catholic University of Chile, Av. Vicuña Mackenna 4860, 7820436 Santiago, Chile; 2 Institute for Mathematical and Computational Engineering, Pontifical Catholic University of Chile, Av. Vicuña Mackenna 4860, 7820436 Santiago, Chile; 3 School of Mathematical Sciences and Centre for Data Science, Queensland University of Technology, 2 George Street, Brisbane, Australia; 4 Australian Institute for Bioengineering and Nanotechnology, The University of Queensland, Building 75, Cnr College Rd and Cooper Rd, Brisbane, Australia; 5 The Novo Nordisk Foundation Centre for Biosustainability, Technical University of Denmark, Building, Kemitorvet 220, 2800 Kongens Lyngby, Copenhagen, Denmark

**Keywords:** Loopless flux, Non-convex space, Genome-scale metabolic models, Monte Carlo methods

## Abstract

**Background:**

Uniform random sampling of mass-balanced flux solutions offers an unbiased appraisal of the capabilities of metabolic networks. Unfortunately, it is impossible to avoid thermodynamically infeasible loops in flux samples when using convex samplers on large metabolic models. Current strategies for randomly sampling the non-convex loopless flux space display limited efficiency and lack theoretical guarantees.

**Results:**

Here, we present LooplessFluxSampler, an efficient algorithm for exploring the loopless mass-balanced flux solution space of metabolic models, based on an Adaptive Directions Sampling on a Box (ADSB) algorithm. ADSB is rooted in the general Adaptive Direction Sampling (ADS) framework, specifically the Parallel ADS, for which theoretical convergence and irreducibility results are available for sampling from arbitrary distributions. By sampling directions that adapt to the target distribution, ADSB traverses more efficiently the sample space achieving faster mixing than other methods. Importantly, the presented algorithm is guaranteed to target the uniform distribution over convex regions, and it provably converges on the latter distribution over more general (non-convex) regions provided the sample can have full support.

**Conclusions:**

LooplessFluxSampler enables scalable statistical inference of the loopless mass-balanced solution space of large metabolic models. Grounded in a theoretically sound framework, this toolbox provides not only efficient but also reliable results for exploring the properties of the almost surely non-convex loopless flux space. Finally, LooplessFluxSampler includes a Markov Chain diagnostics suite for assessing the quality of the final sample and the performance of the algorithm.

## Background

Uniform random sampling has become a useful tool for exploring the solution space of metabolic models in the absence of optimality criteria under different conditions [1]. For a given metabolic model, the solution space is described by a set of equality (mass balances) and inequality (thermodynamic and/or kinetic capacity) constraints. Let us denote by ${{\textbf{S}}} \in \mathbb{R}^{m \times n}$ the stoichiometric matrix of the network, **lb** and **ub** $\in \mathbb{R}^{n}$ the minimum and maximum capacity constraints, respectively, and ${{\textbf{v}}} \in \mathbb{R}^{n}$ a flux solution vector, where *n* denotes the number of reactions and *m* the number of balanced metabolites. Then, the mass-balanced solution space $\varvec{\Omega }$ can be defined as $\varvec{\Omega } = \{{{\textbf{v}}} \ |\ {{\textbf{S}}} \cdot {{\textbf{v}}} = {{\textbf{0}}},\ {\textbf{lb}} \le {{\textbf{v}}} \le {\textbf{ub}}\}$. Importantly, $\varvec{\Omega }$ defines a convex body (polytope) on $\mathbb{R}^{n - \text{rank} \left( {{\textbf{S}}}\right) }$ that can be uniformly sampled and readily explored using Monte Carlo methods, e.g., Hit-and-Run (HR) [2]. Although HR theoretically converges to the uniform distribution on $\varvec{\Omega }$, its implementation in practice is difficult given the highly heterogeneous scales spanned in metabolic models. To overcome this limitation, different approaches have been proposed for improving its performance [3–5]. For instance, the Artificial Centering Hit-and-Run (ACHR) displays accelerated mixing by sampling elongated directions enabling longer steps [5]; unfortunately, the sequence of iterates does not describe a Markov chain, and hence, it is not assured to converge to the target distribution. More recently, the Coordinate Hit-and-Run with Rounding (CHRR) [4] algorithm has been proposed to overcome flux ill-conditioning while maintaining convergence guarantees. Briefly, by computing a maximum volume ellipsoid inscribed in the heterogeneous polytope, the original space can be rounded into a unit ball that can be readily sampled using tractable HR algorithms like the Coordinate Hit-and-Run (CHR) [2]. To this date, this implementation remains the most consistent and efficient method for sampling the mass-balanced flux solution space of genome-scale metabolic models [6], enabling statistical inference of the metabolic phenotype of single- [7, 8] as well as multi-cellular organisms [9, 10], and even microbial communities [11].

A more challenging problem consists of producing a uniform mass-balanced “loopless” flux solution sample from a metabolic model. Flux solutions with active closed loops are not only unrealistic and obscure statistical inference [12], but violate a “loop law” that is analogous to Kirchhoff’s second law for electrical circuits [13, 14]. Any steady-state flux distribution ${{\textbf{v}}}$ can be expressed as ${{\textbf{v}}} = {{\textbf{v}}}_{\text{ll}} + \Delta {{\textbf{v}}}$, where ${{\textbf{v}}}_{\text{ll}}$ denotes a loopless flux distribution and $\Delta {{\textbf{v}}}$ represents an internal flux distribution (i.e., without active exchanges). The latter flux distributions are both mass-balanced and can be written as [15],

1 $$\begin{aligned} {{\textbf{v}}}_{\text{ll}}&= \sum _{i} \alpha _i \cdot {{\textbf{e}}}_{i}^{(\text{I})} \nonumber \\ \Delta {{\textbf{v}}}&= \sum _{j} \beta _j \cdot {{\textbf{e}}}_{j}^{(\text{III})} \end{aligned}$$

where ${{\textbf{e}}}_{i}^{(\text{I})}$ and ${{\textbf{e}}}_{j}^{(\text{III})}$ represent type I (pathways with a net conversion of substrates to products) and type III (internal cycles without net conversion) extreme pathways, respectively [16]. In Eq. (1) ${{\textbf{v}}}_{\text{ll}}$ and $\Delta {{\textbf{v}}}$ are convex combinations of the respective flux modes. Notably, the internal flux distribution fulfills ${{\textbf{S}}}_{\text{int}} \cdot \Delta {{\textbf{v}}}_{\text{int}} = {{\textbf{0}}}$, where “int” refers to the set of internal reactions. Then, the loopless mass-balanced flux solution space can be defined as $\varvec{\Omega }_{\text{loopless}} = \{{{\textbf{v}}} \ |\ {{\textbf{S}}} \cdot {{\textbf{v}}} = {{\textbf{0}}},\ {\textbf{lb}} \le {{\textbf{v}}} \le {\textbf{ub}},\ \Delta {{\textbf{v}}} = {{\textbf{0}}} \text{ and } {{\textbf{v}}} \ne {{\textbf{0}}}\}$. $\varvec{\Omega }_{\text{loopless}}$ describes almost surely a non-convex space that is substantially harder to sample from. To tackle this challenge, we previously have proposed an approximate algorithm based on ACHR termed loopless Artificial Centering Hit-and-Run on a Box (ll-ACHRB), which yields a (non-uniform) random sample from $\varvec{\Omega }_{\text{loopless}}$ in reasonable time [17]. In this work, we present a superior algorithm termed Adaptive Direction Sampling on a Box (ADSB) for generating a provably uniform random flux sample on $\varvec{\Omega }_{\text{loopless}}$ with substantially higher computational performance and stronger theoretical support.

## Implementation

### Algorithm

**Fig. 1:**
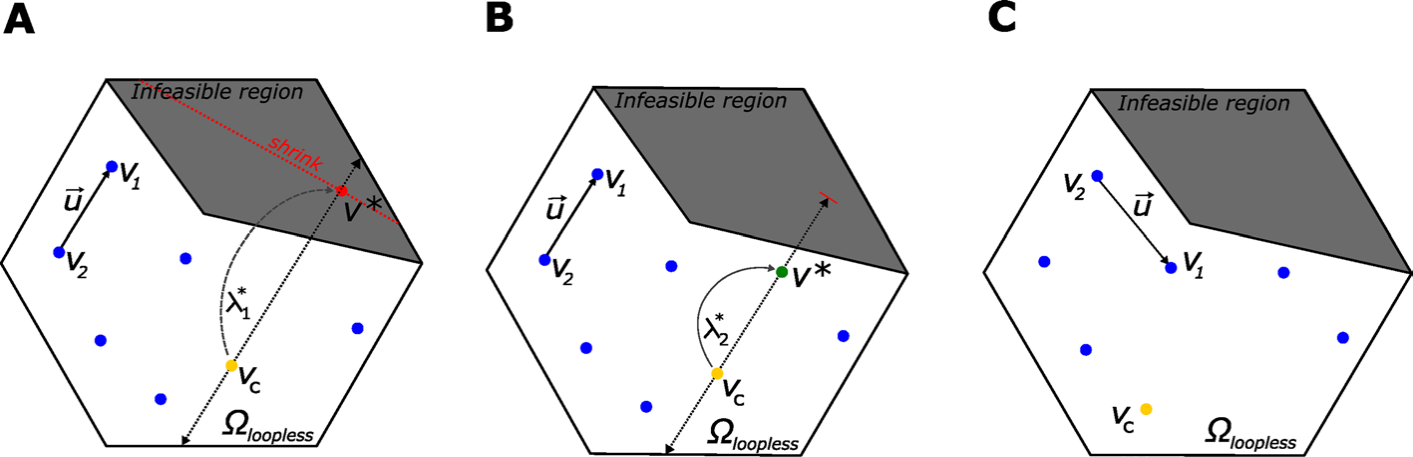
Illustration of the ADSB algorithm. **A** Consider a current set **V**$^{(t)}$ composed of $k = 7$ points. At each step, **v**$_{c}$ and two other points (**v**$_{1}$,**v**$_{2}$) are randomly drawn from **V**$^{(t)}$ and used to construct the direction of movement **u**$^*$. A uniform random step $\lambda ^*$ is taken along a cord passing through **v**$_{c}$ and parallel to **u**$^{*}$. A new point **v**$^{*} =$ **v**$_{c} + \lambda ^*$ **u**$^{*}$ is proposed. If **v**$^{*} \notin \varvec{\Omega }_{\text{loopless}}$, then **v**$^*$ is rejected and the initial cord is shrunk. **B** The length of the initial cord is shrunk and a new step is taken. This time the new point **v**$^*$ lands on a feasible region and it is therefore accepted. **C** Once a feasible point is found, **v**$^{*}$ is swapped for **v**$_{c}$ in the current set **V**$^{(t+1)}$ and the algorithm is repeated

ADS belongs to a family of population-based MCMC methods that involve sampling from an augmented $k \times \text{dim}(\Theta )$-dimensional state space over support($\Theta$). At each iteration, ADS maintains a set of *k* points (vectors) on $\Theta$ in a current set ${{\textbf{V}}}^{(t)}$ that is used to adaptively construct convenient directions for traversing support($\Theta$) according to the target distribution $\pi ({{\textbf{v}}})$ [18]. In this case, $\pi ({{\textbf{v}}})$ is the uniform distribution over $\varvec{\Omega }_{\text{loopless}}$ (previously $\Theta$), which is spanned by *k* mass-balanced loopless flux vectors contained in ${{\textbf{V}}}^{(t)}$. Particularly in parallel ADS, a new point ${{\textbf{v}}}^{*}$ is proposed by sampling on the line ${\mathcal {L}}\{ \}$ passing through a random point ${{\textbf{v}}}_{c}^{(t)} \in {{\textbf{V}}}^{(t)}$ (current point), and parallel to a direction ${{\textbf{u}}}^{*}$ constructed from two distinct random points (${{\textbf{v}}}_1^{(t)},{{\textbf{v}}}_2^{(t)}$) from ${{\textbf{V}}}^{(t)}$ [18] (Fig. 1A). A uniform random step $\lambda ^{*}$ is then drawn on the cord ${\mathcal {L}}({{\textbf{v}}}_{c}^{(t)} +\lambda {{\textbf{u}}}^{*})$, and ${{\textbf{v}}}_c^{(t)}$ is replaced by ${{\textbf{v}}}^{*}$ in ${{\textbf{V}}}^{(t+1)}$ leaving all other points unchanged [18]. In this way, the parallel ADS algorithm produces a reversible Markov chain ${{\textbf{V}}} = \{{{\textbf{V}}}^{(t)}, t \in \mathbb{Z}^{+}\}$ in the augmented state space according to the uniform distribution on $\varvec{\Omega }_{\text{loopless}}$ as long as $\text{dim}({{\textbf{V}}}^{(t)}) = \text{dim}(\varvec{\Omega }_{\text{loopless}})$ [19]. Direct application of ADS to our case is, however, not possible as the actual support $\varvec{\Omega }_{\text{loopless}}$ is likely non-convex, which renders sampling $\lambda ^{*}$ hard. To overcome this obstacle, ADSB borrows the “shrinking box” method from slice sampling [20] to efficiently propose new points on $\varvec{\Omega }$ and retaining those in $\varvec{\Omega }_{\text{loopless}}$ [21] (Fig. 1B, C). To check whether a flux vector is loopless, the method proposed in [17] for loop detection was employed, which is based on the fact that active closed loops can be *topologically* detected directly from the sign pattern of a given flux vector [22]. Importantly, as long as ${{\textbf{V}}}^{(t)}$ spans $\varvec{\Omega }_{\text{loopless}}$, or equivalently, as long as any ${{\textbf{v}}} \in \varvec{\Omega }_{\text{loopless}}$ can be reached from any ${{\textbf{v}}}^{(t)} \in {{\textbf{V}}}^{(t)}$ [23], the asymptotic convergence to the arbitrary target (uniform) distribution is guaranteed [24]. Finally, the computational performance of ADSB is improved by running *K* non-interacting Markov chains in parallel each with *k* points in ${{\textbf{V}}}$. Details of the proposed algorithm such as pre-processing, selection of the initial points, number of iterations and the pseudo-code can be found in the Additional file 1.

### Computational implementation

LooplessFluxSampler is implemented in MATLAB (Mathworks, Natick, MA) with an interface to the popular COBRA Toolbox [25], enabling ready application to constraint-based metabolic models. The software runs in Windows, macOS and Linux, on machines with a 64-bit CPU architecture. Vectorized mathematical code has been employed to speed up parallel computations. LooplessFluxSampler makes use of the Parallel Computing Toolbox from MATLAB to speed computations, albeit the latter is not strictly necessary for single-core computations. For sampling large models, this implementation can take advantage of multiple cores with an almost linear scaling when the Parallel Computing Toolbox is available.

LooplessFluxSampler requires the user to provide the metabolic model in COBRA format (.MAT structure), along with the number of samples to be generated. Additional file 1 such as burn-in, thinning, number of cores, sampler (ADSB, ll-ACHRB, and HR), warmup, among others, can be provided if desired. Before sampling, metabolic models are pre-processed to remove blocked reactions using fast-SNP for fast loopless flux optimization [26]. After the sampling, a complete Markov chain diagnostics can be run to assess the quality of the final sample and the performance of the algorithm. Finally, the results presented here were obtained on a 16-CPU 64-GB ram Virtual Machine hosted in the QRIScloud Polaris cell.

## Results and discussion

The performance of ADSB is demonstrated in two scenarios assessing different capabilities. First, we evaluated the convergence behavior on the uniform distribution in a core metabolic model of *E. coli* [27], where flux solutions have *full support* [28]. Due to the loopless condition imposition, reactions can only carry flux *conditional* on the fluxes through other reactions. In some networks, all reactions cannot simultaneously carry flux without violating the loopless condition [17], hindering full exploration of $\varvec{\Omega }_{\text{loopless}}$ by ADSB. In such cases, only a subspace of $\varvec{\Omega }_{\text{loopless}}$ can be uniformly sampled as some reactions will be necessarily blocked. Thus, we initially assessed the performance of ADSB in metabolic networks where $\varvec{\Omega }_{\text{loopless}}$ remained *conditionally* full-dimensional. For this task, four synthetic networks were analyzed using the model with the original bound (capacity) constraints, but forcing a different number of irreversible reactions to be reversible (Additional file 1). As a consequence, the number of potentially active closed loops in the synthetic networks increased, rendering the sampling task more challenging. The resulting models contained 0, 2, 10 and 12 potentially active loops while enabling all their reactions to carry simultaneously flux.

**Fig. 2:**
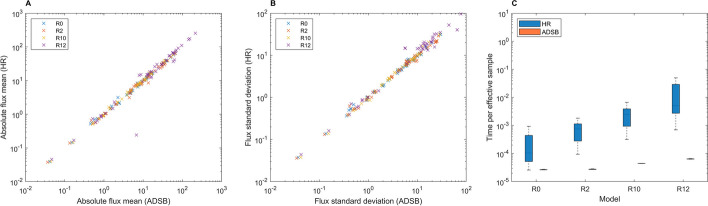
Convergence comparison of ADSB and HR in *E. coli* core metabolic models with varying number of potentially active closed loops. Correlation between flux means (**A**) and standard deviations (**B**) between both samplers in all models. Synthetic models R0, R2, R10 and R12 contain 0, 2, 10 and 12 potentially active closed loops, respectively. The reactions forced to be reversible to generate these models can be found in Section 6 of the Additional file 1. **C** Comparison of the convergence behavior of ADSB and HR. Each model was simulated on minimal medium growing aerobically. Both algorithms were run under the same conditions: $2 \cdot 10^{5}$ final points, 100 steps per point (thinning), and first $2 \cdot 10^{4}$ points discarded (burn-in)

The convergence behavior of ADSB was compared against HR considering that: (1) HR is proven to converge in total variation to a uniform distribution on convex regions [2], and (2) points from HR are in $\varvec{\Omega }$ and can be later verified to be in $\varvec{\Omega }_{\text{loopless}}$ by checking if they are loopless. To ensure a fair comparison, both ADSB and HR were initialized using over-dispersed seeds without warmup. Then, if ADSB produces a sample with similar characteristics to HR, there is a strong support for its convergence to the uniform distribution. This was indeed the case (Fig. 2). Overall, the sample means (Fig. 2A) and standard deviations (Fig. 2B) of all the fluxes showed high consistency and no significant statistical difference was detected (adjusted $p\text{-value}>0.05$, Wilcoxon signed-ranked test, refer to Additional file 1: Table S3 and Figure S1). There was only one instance in model 12 (NADH dehydrogenase reaction, ID: NADH16), where a flux mean differed appreciably between samplers. Subsequent inspection of this case revealed that HR mixed less efficiently in this particular variable, which explained the observed difference (potential scale reduction factor (*psrf*) $\approx$ 1.053 vs. 1.00 of ADSB, Additional file 1: Figure S2). Importantly, in terms of sampling performance, ADSB consistently displayed reduced time per effective sample (Fig. 2C) and *psrf* very close to 1 and with lower variability than HR (Additional file 1: Figure S3). In fact, for models with a higher number of potentially active closed loops, HR displayed higher time per effective sample and hence a lower performance than ADSB (Fig. 2C). Altogether, the above results support the convergence behavior of ADSB and a superior performance for more complex models than the theoretically proven HR.

**Fig. 3:**
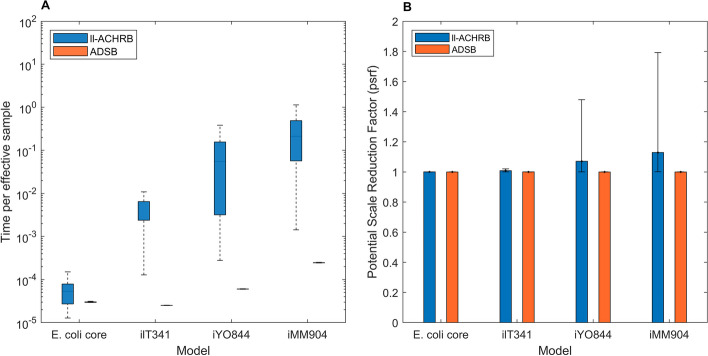
Performance comparison of ADSB and ll-ACHRB in different metabolic models. **A** Sampling performance and **B** mixing behavior were evaluated in ADSB and ll-ACHRB in large metabolic models. Each model was simulated on aerobic minimal medium (default simulation conditions). Both algorithms were run using the settings: $2 \cdot 10^{5}$ final points, 100 steps per point (thinning), and first $2 \cdot 10^{4}$ points discarded (burn-in)

Next, we evaluated the computational performance and scalability of ADSB by comparing its performance against ll-ACHRB. Again, to ensure a fair comparison between samplers, both ll-ACHRB and ADSB were initialized using over-dispersed seeds determined through optimization without warmup. For this task, large metabolic models spanning different sizes were chosen (Fig. 3). In these models, all reactions could not carry flux *simultaneously* without violating the loopless condition, and thus, there is no guarantee of convergence to the uniform distribution for the full model. We note that this guarantee could be recovered for a submodel made of only flux-carrying reactions.

Previous results have shown that in such complex models, i.e., with various potentially active loop laws, it is infeasible to generate a loopless flux sample without blocking specific reactions [17]. In these cases, a strategy like the previously employed in the *E. coli* core model will be extremely inefficient at producing loopless flux samples. An alternative is to formulate and solve an optimization problem where infeasible loops are removed from flux samples after uniform random sampling [29]. However, even if efficient cycle removal formulations are used (see e.g., CycleFreeFlux [30]), the computation time will be substantial as various thousands of flux samples are typically drawn for sampling analyses. More importantly, this random flux sample has no guarantee of targeting the uniform distribution. This background further supports the need of loopless flux samplers for analysing large metabolic models.

ADSB displayed from an approximately 4-fold increased average computational performance in the small *E. coli* core model, up to an approx. 1000-fold increased performance in the large iMM904 model when compared to ll-ACHRB (Fig. 3A, Additional file 1: Table S4). The computational performance was measured as the running time divided by the average (over the fluxes) effective sample size $N_{\text{eff}}$ (the number of independent samples the final correlated sample is worth), which is a fair indicator of the sample quality. Overall, ADSB was substantially superior to ll-ACHRB achieving two orders of magnitude increased computational performance in the smallest genome-scale model (see iIT341, Fig. 3A). Moreover, when comparing mixing properties, only ADSB showed a consistent behavior across all the tested models ($psrf \approx 1$, Fig. 3B). This was not the case for ll-ACHRB in the larger models iYO844 and iMM904 (Fig. 3B). Importantly, ADSB displayed low *psrf* variability, which points to a consistent and scalable sampling performance in larger models.

## Conclusions

LooplessFluxSampler provides an efficient tool for randomly sampling the loopless flux solution space of large-scale metabolic models, displaying both superior computational performance and convergence behavior than the alternatives. Under conditions where the loopless flux solution space can be described by flux vectors with full support, this tool provably converges on the uniform distribution ensuring consistent results. Finally, the LooplessFluxSampler is fully compatible with the popular COBRA Toolbox, enabling rapid adoption by modelers and practitioners in the field. We expect to deploy this software on other open-source platforms such as Python and Julia in the near future to expand its use by the scientific community.

### Supplementary Information

**Additional file 1.** Additional information describing algorithmic details and results of the application of the sampler in benchmark cases.

## Data Availability

The LooplessFluxSampler function library along with example codes are freely available via the GitHub repository for this project: • Project name: LoopessFluxSampler. • Project home page (including examples of use): https://github.com/SysBioengLab/looplessFluxSampler • Operating system: Platform independent. • Programming language: MATLAB environment. • Other requirements: COBRA Toolbox v3.0. • License: MIT. • Any restrictions to use by non-academics: None.
