# Supplementary material for: LooplessFluxSampler: an efficient toolbox for sampling the loopless flux solution space of metabolic models
Source: BMC Bioinformatics. 2024 Jan 2;25:3. doi: 10.1186/s12859-023-05616-2 (PMC10763395; doi:10.1186/s12859-023-05616-2)
Supplement: Supplementary file 1 — Additional file 1. Additional information describing algorithmic details and results of the application of the sampler in benchmark cases. [file 12859_2023_5616_MOESM1_ESM.pdf]

Supplementary Information
LooplessFluxSampler: An efficient toolbox for sampling the loopless flux solution space of metabolic models

Pedro A. Saa, Sebastian Zapararte, Christopher C. Drovandi, Lars K. Nielsen

December 12, 2023

Contents

1 About this document 3

2 Loopless flux sampler workflow 3

3 The Adaptive Direction Sampling framework 3

4 Determination of the iteration number 6

5 Implementation of ADSB on $\Omega_{\mathbf{loopless}}$ 7

6 Benchmark against reported samplers 7

List of Figures

S1 Comparison of flux means and standard deviations distributions between HR and ADSB. **(A)** Scaled flux mean differences between HR and ADSB. **(B)** Scaled flux standard deviation differences between HR and ADSB. In both cases, flux means and standard deviation were scaled by the corresponding reaction range. 9
S2 Comparison of the marginal distributions for the NADH16 reaction (NADH dehydrogenase) obtained with HR and ADSB. The potential scale reduction factor (*psrf*) for HR was 1.053 whereas as for ADSB was 1.00. 10
S3 Comparison of the Potential Scale Reduction Factor (*psrf*) for HR and ADSB in various versions of the *E. coli* core model. 11

List of Tables

S1 Average relative difference in reaction fluxes means between HR and parallel ADS under different settings in the *E. coli* core model 6
S2 Average relative difference in reaction fluxes standard deviations between HR and parallel ADS under different settings in the *E. coli* core model 6
S3 Correlation between statistics from HR and ADSB 8
S4 Sampling performance of ADSB and ll-ACHRB in different metabolic models 9

List of Algorithms

1 Loopless Flux Sampler 4
2 Shrinking method for ADSB 7

1 About this document

This Supplementary Material presents details and additional performance results of the proposed Adaptive Direction Sampling on a Box (ADSB) algorithm for sampling the loopless, mass-balanced flux solution space of metabolic models. The proposed algorithm has been implemented in MATLAB 2021a (Mathworks, Natick, MA) and it has been successfully run up to MATLAB version 2014b in both Windows and Linux OS. This submission also includes illustrative examples describing how ADSB works on tests cases. The MATLAB code is open source and can be downloaded from https://github.com/SysBioEngLab/looplessFluxSampler. Finally, for questions related to the operation/performance of the algorithm and/or for reporting issues/bugs, please contact pnsaa@ing.puc.cl.

2 Loopless flux sampler workflow

The general workflow of the proposed algorithm is shown in the Algorithm 1. Starting from a COBRA model defined by the stoichiometric matrix ($\mathbf{S}$) and flux bounds ($\mathbf{lb}$ and $\mathbf{ub}$), the loopless flux sampler generates a loopless flux sample of size N and a set of MCMC diagnostics statistics to assess the quality of the sample (optional). The final diagnostics phase is run by default, although it can be disabled if desired. This is however not recommended as assessment of the sample quality is critical for performing rigorous statistical inference.

The sampling workflow consists of three phases, namely: 1) Pre-processing, 2) Warmup, and 3) Sampling. In the first phase, all blocked reactions – either due to mass balances or cycle-free flux constraints – are removed using loopless Flux Variability Analysis [1]. Removal of these reactions is critical to ensure the appropriate performance of the sampler, as maintaining zero reactions during the sampling inevitably leads to numerical and performance issues in large metabolic models (e.g., generation of effective directions of movement becomes hard) [2, 3]. Once the model has been pre-processed, an iterative warmup phase takes place where an initial sample seed is generated ($warmupPoints$) using ll-ACHRB [3]. As the latter will be used to construct the current set $\mathbf{V}^{(t)}$, ensuring high quality of this sample is critical to achieve good performance later on. Here, we employed the Potential Scale Reduction Factor ($psrf$) of the seed's centroid (R_{centroid}) as an indicator of the convergence, and hence, quality of the seed. Briefly, in each warmup iteration, the seed number is doubled until the convergence criterion $R_{\text{centroid}} \in [0.9, 1.1]$ is achieved ($R_{\text{centroid}} \approx 1$ suggests good mixing, [4]). Once this criterion is met, k random points ($k \geq \dim(\Omega_{\text{loopless}})$) are sampled without replacement from the warmup seeds N_{chains} times and assigned to N_{chains} current sets. In this way, if N_{points} denotes the size of the desired sample, then one has $k \cdot N_{\text{chains}} = N_{\text{points}}$. The number k of points is defined relative to $\dim(\Omega_{\text{loopless}})$ (see section below). Finally, the initialized current sets will be part of N_{chains} non-interacting Markov Chains running the ADSB algorithm in parallel. The details of the general loopless flux sampler workflow are shown in Algorithm 1, whereas the details of the ADSB sampler are shown in the next section.

3 The Adaptive Direction Sampling framework

The general ADS algorithm encompasses a number of known MCMC methods for generating samples $\mathbf{v}$ according to a target distribution $\pi(\mathbf{v})$ on the support Θ. In the following, we will describe the general ADS from which the special parallel ADS is derived. Let M be (k–1) sets of n-tuples, where n equals $\dim(\Theta)$, or, in our case, the degrees of freedom of the metabolic model. Then, for all $m \in M$, let $D_r(m)$ and $D_u(m)$ be distributions on $\mathbb{R}$ and $\mathbb{R}^n$, respectively. Choose $\mathbf{V}^{(0)}$ as the

Algorithm 1: Loopless Flux Sampler

Inputs : Constraint-based metabolic model (COBRA model structure defined by fields **S**, **lb** and **ub**), and number of random flux samples N_{points}

Outputs: N_{points} loopless mass-balanced flux points drawn from Ω_{loopless} ($vPoints$), MCMC diagnostics convergence summary statistics (optional).

Pre-processing phase
$constrainedModel \leftarrow runFVA(model)$
$looplessConstrainedModel \leftarrow runLooplessFVA(constrainedModel)$
$reducedModel \leftarrow removeBlockedReactions(looplessConstrainedModel)$
Warmup phase
$warmupPoints \leftarrow \oslash$
$numWarmupPoints \leftarrow 2 \cdot 10^4$ (default)
$maxWarmupPoints \leftarrow 2 \cdot 10^6$ (default)
$R_{\text{centroid}} \leftarrow \infty$
while $|R_{centroid} - 1| > 0.1$ *and* $numWarmupPoints < maxWarmupPoints$ **do**
  $vWarmup \leftarrow runllACHRB(reducedModel, numWarmupPoints)$
  $warmupPoints \leftarrow append(warmupPoints, vWarmup)$
  $centroidTrace \leftarrow computeCentroidTrace(warmupPoints)$
  $R_{\text{centroid}} \leftarrow computePotentialScaleReductionFactor(centroidTrace)$
  $numWarmupPoints \leftarrow 2 \cdot numWarmupPoints$
end
Initialize $k \geq \dim(\Omega_{\text{loopless}})$ and set $N_{\text{chains}} \leftarrow round(N_{\text{points}}/k)$
foreach $j \in \{1, ..., N_{chains}\}$ **do**
  $currentSet_j \leftarrow sampleWithoutReplacementKpoints(warmupPoints)$
end
Sampling Phase
foreach $parallelMarkovchainj$ **do**
  $vPointsChain_j \leftarrow runADSB(reducedModel, currentSet_j)$
end
Set $vPoints \leftarrow \oslash$ and Append $vPointsChain_j$ to $vPoints$ for $j \in \{1, ..., N_{\text{chains}}\}$
Diagnostics Phase (optional)
$mcmcDiagnostics \leftarrow runMCMCconvergenceDiagnostics(reducedModel, vPoints)$

initial *current set* as defined previously. Given $\mathbf{V}^{(t)}$ for $t \in \mathbb{Z}^+$, ADS proceeds as follows [5, 6].

Step 1. Sample uniformly a random point $\mathbf{v}_c^{(t)}$ from $\mathbf{V}^{(t)}$. This point will be called the *current point*. Let $\mathbf{C}^{(t)} = \mathbf{V}^{(t)} - \{\mathbf{v}_c^{(t)}\}$.

Step 2. Generate r^* from $D_r(\mathbf{C}^{(t)})$ and $\mathbf{u}^*$ from $D_u(\mathbf{C}^{(t)})$.

Step 3. Sample $\lambda^* \in \mathbb{R}$ according to the density on Θ,

$$f(\lambda) \propto |1 + \lambda r^*|^{n-1} \pi(\mathbf{v}_c^{(t)} + \lambda\{r^* \mathbf{v}_c^{(t)} + \mathbf{u}^*\})$$

And let $\mathbf{v}^* = \mathbf{v}_c^{(t)} + \lambda^*(r^* \mathbf{v}_c^{(t)} + \mathbf{u}^*)$

Step 4. Update the current set as $\mathbf{V}^{(t+1)} = \mathbf{V}^{(t)} - \{\mathbf{v}_c^{(t)}\} \cup \{\mathbf{v}^*\}$

The general ADS produces a reversible Markov chain in the augmented $k \times \dim(\Theta)$-dimensional state space that targets the desired distribution $\pi(\mathbf{v})$. A number of special cases can be identified for different choices of r^* and $\mathbf{u}^*$.

i) If $r^* = -1, \mathbf{u}^* = \mathbf{v}_i^{(t)}$ with probability $(k-1)^{-1}, 1 \leq i \leq (k-1)$ for $\mathbf{v}_i^{(t)} \in \mathbf{C}^{(t)}$, then ADS yields the snooker algorithm for which powerful irreducibility results can be derived under extremely mild regularity conditions [6]. The cost of this attractive sampler is that at each iteration one has to sample from the density $f(\lambda) \propto |1 - \lambda|^{n-1}\ \pi(\mathbf{v}_c^{(t)}(1 - \lambda) + \mathbf{u}^*)$, which may be improper and/or more expensive to sample from.

ii) If one defines a fixed set of coordinate directions $\mathcal{T} = \{d_1, ..., d_n\}$, set $r^* = 0$, and let $\mathbf{u}^*$ be chosen uniformly at random from $\mathcal{T}$, this yields a Gibbs sampler (similar to the Coordinate Hit-And-Run) [7].

iii) Set $r^* = 0$, and let $\mathbf{u}^*$ be chosen uniformly from the surface of the unit hyper-sphere in $\mathbb{R}^n$, this yields the general Hit-And-Run (HR) [8].

iv) Set $r^* = 0$, and let $\mathbf{u}^* = \mathbf{v}_1^{(t)} - \mathbf{v}_2^{(t)}$, where $\mathbf{v}_1^{(t)}$ and $\mathbf{v}_2^{(t)}$ are distinct and randomly chosen points from $\mathbf{C}^{(t)}$. This is the parallel ADS algorithm [5].

As previously noted, the ADS framework can produce different samplers targeting the sought distribution by conveniently selecting D_r and D_u. Notably, by choosing $r^* = 0$ (cases ii, iii and iv), and considering that in our target distribution is the uniform distribution, Step 3 of ADS can be greatly simplified and reduced to uniformly sampling on a line. Of all the possible choices, the parallel ADS looks the most promising as it dynamically stores information about $\pi(\mathbf{v})$ over Θ as opposed to the Gibbs and Hit-And-Run samplers.

To test the suitability of this sampler, we compared the convergence properties of this algorithm against the popular Hit-And-Run using current sets of different sizes (k). As a benchmark, we sampled from the *E. coli* core model without enforcing the loopless condition. In the case of the HR, we produced $2 \cdot 10^5$ samples with a thinning factor of 200 steps and discarding the first 10^5 samples. We will consider the resulting marginals from this sample the gold standard for comparison.

Table S1: Average relative difference in reaction fluxes means between HR and parallel ADS under different settings in the *E. coli* core model

Points in current set $\mathbf{V}^{(t)}$	$i = 100$	$i = 200$	$i = 300$
$k = n + 1$	10.8%	9.2%	6.9%
$k = 2n$	10.1%	7.7%	4.8%
$k = 3n$	9.7%	7.4%	4.4%

Table S2: Average relative difference in reaction fluxes standard deviations between HR and parallel ADS under different settings in the *E. coli* core model

Points in current set $\mathbf{V}^{(t)}$	$i = 100$	$i = 200$	$i = 300$
$k = n + 1$	10.5%	9.9%	8.1%
$k = 2n$	9.9%	8.9%	6.6%
$k = 3n$	9.7%	8.3%	6.0%

Tables S1 and S2 summarize the average relative difference between the main statistics from a HR sample and a sample of size $2 \cdot 10^4$ generated from the parallel ADS under different settings.

Overall, our results indicate that the parallel ADS performs satisfactorily, displaying good consistency with HR despite using a sample 10 times smaller. Most notably, the parallel ADS improves its performance substantially with increasing number of points in $\mathbf{V}^{(t)}$. In the following, we present a convenient heuristic for determining a suitable number of iterations for running the algorithm.

4 Determination of the iteration number

At each iteration, the parallel ADS chooses $\mathbf{v}_c^{(t)}$ (current point) randomly from $\mathbf{V}^{(t)}$ (current set) and proposes a new point $\mathbf{v}^*$ using this information and an appropriate direction $\mathbf{u}^*$. For a valid $\mathbf{v}^*$ on Θ, $\mathbf{v}^*$ replaces $\mathbf{v}_c^{(t)}$ in $V^{(t)}$ (Step 4) and the algorithm proceeds. As shown previously, the more the current set is updated with new points, the faster it will converge on the target (uniform) distribution.

In our case, we would like to move each point in $\mathbf{V}^{(t)}$ at least $n_{\min}$ times with some high probability p_{move}, say 0.99. Considering that the probability p of choosing a point from $\mathbf{V}^{(t)}$ is constant ($p = 1/k$) and that this represents a Bernoulli trial, then the number of iterations n_{move} required for moving each point at least $n_{\min}$ times with probability p_{move} can be calculated using the Binomial distribution.

$$P(X \geq n_{\min} \mid n_{\text{move}}, p) = p_{\text{move}}$$

$$p_{\text{move}} = 1 - P(X \leq n_{\min} - 1 \mid n_{\text{move}}, p)$$

$$p_{\text{move}} = 1 - \sum_{j=0}^{n_{\min}-1} \binom{n_{\text{move}}}{j} \cdot p^j \cdot (1-p)^{n_{\text{move}}-j}$$

$$\sum_{j=0}^{n_{\min}-1} \binom{n_{\text{move}}}{j} \cdot p^j \cdot (1-p)^{n_{\text{move}}-j} = 1 - p_{\text{move}}$$

The last equation can be approximately solved numerically for n_{move} provided p_{move}, $n_{\min}$, and $p = k^{-1}$. By default ADSB uses $p_{\text{move}} = 0.99$ and $n_{\min} = 500$.

5 Implementation of ADSB on Ω_{loopless}

The key challenge in the implementation of the parallel ADS method for sampling the loopless, mass-balanced flux solution space stems from the difficulty of proposing new points inside Ω_{loopless}. Indeed, as Ω_{loopless} is almost surely non-convex, one cannot know for certain if a proposal $\mathbf{v}^* \in \Omega_{\text{loopless}}$ even if $\mathbf{v}^* \in \Omega$. We note, however, that $\Omega_{\text{loopless}} \subseteq \Omega$, so one can readily propose $\mathbf{v}^*$ on Ω and reject if $\mathbf{v}^*$ violates the loopless condition [3]. If the proposal is rejected, this step can be repeated with a new proposal until $\mathbf{v}^* \in \Omega_{\text{loopless}}$. This rejection method is however very inefficient. A more efficient method for proposing on non-convex regions has been reported in the context of slice sampling [9]. After each rejection, this method shrinks the 'enclosing box' (defined by the intersection of Ω and the line through $\mathbf{v}^*$) and checks whether a new proposal on the 'shrunk' space is feasible. Computationally, this method is superior to rejecting new proposals from Ω. Importantly, this method has been shown to generate samples according to the uniform distribution using HR in non-convex regions [10]. The shrinking Algorithm is shown below.

Algorithm 2: Shrinking method for ADSB

Inputs : Current point $\mathbf{v}_c$, lower and upper flux bounds ($\mathbf{lb}$,$\mathbf{ub}$), direction of movement $\mathbf{u}^*$
Outputs: Feasible point $\mathbf{v}^*$

Set $(\lambda^{\max-}, \lambda^{\max+}) \leftarrow \mathit{determineLongestCord}(\mathbf{v}_c, \mathbf{u}^*, \mathbf{lb}, \mathbf{ub})$ and $\mathit{condition} \leftarrow \mathit{true}$
while *condition* **do**
    Sample λ^* uniformly from the interval $(\lambda^{\max-}, \lambda^{\max+})$
    $\mathbf{v}^* \leftarrow \mathbf{v}_c + \lambda^* \mathbf{u}^*$
    if *isLoopless*($\mathbf{v}^*$) **then**
        $\mathit{condition} \leftarrow \mathit{false}$
    else
        if $\lambda^* > 0$ **then**
            Set $\lambda^{\max+} \leftarrow \lambda^*$
        else
            Set $\lambda^{\max-} \leftarrow \lambda^*$
        end
    end
end

Implementation of the above method within the parallel ADS framework enables proposing from Ω_{loopless} with high efficiency. We call the resulting algorithm ADSB because loopless flux solutions are generated using the 'box-shrinking' method.

6 Benchmark against reported samplers

This section complements the analysis presented in the main manuscript, comparing the efficiency and convergence properties of ADSB against HR and ll-ACHRB. For this task, four models of increasing complexity were generated based on the *E. coli* core model reported elsewhere [11]. The FRD7 reaction was removed to enable the generation of mass-balanced, loopless flux samples with *full support* [3]. To generate models with 0 (R0), 2 (R2), 10 (R10), and 12 (R12) potentially active closed loops, reversibility of the following reactions was enabled:

R0: Reversibilities by default.

R2: AKGDH, GLUN, NADTRHD, PPC.

R9: CS, SUCCt2_2, SUCCt3, SUCDi.

R12: GND, NADH16, NADTRHD, PGL.

Once the models were generated, linear regression models were built comparing the similarity between HR and ADSB samples with respect to the flux means and standard deviations. The aim was to verify a consistent convergence behavior of ADSB. The slope and R-squared parameter are reported in Table S3 (see below). The former serves as a measure of similarity between samplers, while the latter indicates how reliable the fit is. In all cases, there were no significant differences in means and standard deviations at a 5% significance level (Wilcoxon rank sum test, Table S3). Estimated p-values were overall high (> 0.9) with the exception of the standard deviation of model R12 (0.229), which was still well above the significance level.

Table S3: Correlation between statistics from HR and ADSB

Model	Slope	R-squared	Adjusted p-value Wilcoxon rank sum test
Mean			
R0	1.003	0.998	0.960
R2	0.988	0.994	0.977
R9	1.014	0.999	0.967
R12	1.021	0.990	0.909
Standard deviation			
R0	0.977	0.996	0.901
R2	0.981	0.996	0.909
R9	1.025	0.997	0.913
R12	1.016	0.984	0.229

Figure S1 confirms that the flux mean and standard deviation differences are centered around zero for both samplers, although greater dispersion is observed for more complex models with higher number of potentially active loops, i.e., R12. Even though for this model there was one reaction with a notable difference in flux means, analysis of the marginals suggests that this is mostly an effect of the HR not mixing as fast as ADSB (Fig. S2). Finally, in order to assess the sampling efficiency of ADSB, its sampling performance was compared in a range of different models against the ll-ACHRB algorithm [3] – the only loopless flux sampler reported to date. The results indicate that ADSB is substantially superior and more robust than ll-ACHRB in range of large-scale metabolic models, reaching up to three orders of magnitude better performance (see iMM904, Table S4).

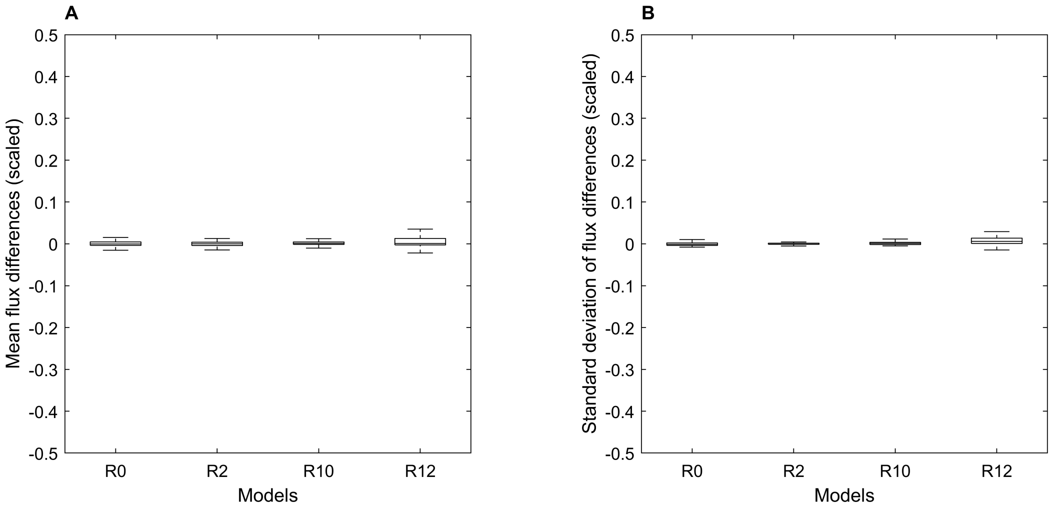

Figure S1: Comparison of flux means and standard deviations distributions between HR and ADSB. **(A)** Scaled flux mean differences between HR and ADSB. **(B)** Scaled flux standard deviation differences between HR and ADSB. In both cases, flux means and standard deviation were scaled by the corresponding reaction range.

Table S4: Sampling performance of ADSB and ll-ACHRB in different metabolic models

Model	Prep. time	Sampling time	Runtime	Median N_{eff}	Median R	Time per effective sample
ll-ACHRB						
E. coli core	8.02E-02	2.88E+02	2.88E+02	6.16E+04	1.000	4.67E-03
*i*IT341	9.05E-01	3.90E+02	3.91E+02	1.92E+05	1.005	2.03E-03
*i*YO844	3.92E+00	8.03E+02	8.07E+02	3.59E+02	1.016	2.25E+00
*i*MM904	9.11E+00	1.60E+03	1.61E+03	2.54E+02	1.036	6.36E+00
ADSB						
E. coli core	1.30E-01	1.08E+02	1.08E+02	1.96E+05	1.000	5.54E-04
*i*IT341	1.41E+00	2.29E+02	2.30E+02	1.95E+05	1.001	1.18E-03
*i*YO844	6.29E+00	1.50E+03	1.50E+03	1.99E+05	1.000	7.54E-03
*i*MM904	1.05E+01	1.20E+03	1.21E+03	2.00E+05	1.000	6.08E-03

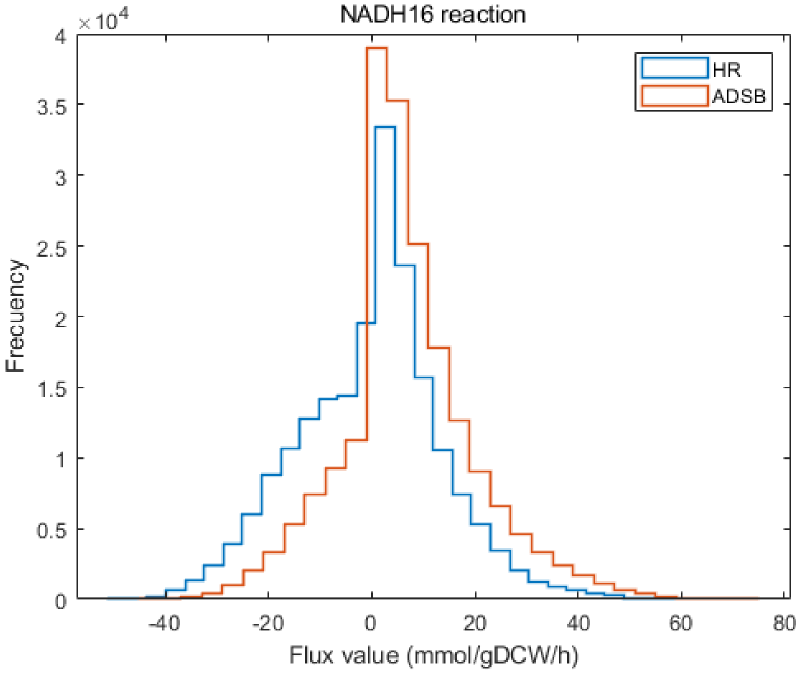

Figure S2: Comparison of the marginal distributions for the NADH16 reaction (NADH dehydrogenase) obtained with HR and ADSB. The potential scale reduction factor ($psrf$) for HR was 1.053 whereas as for ADSB was 1.00.

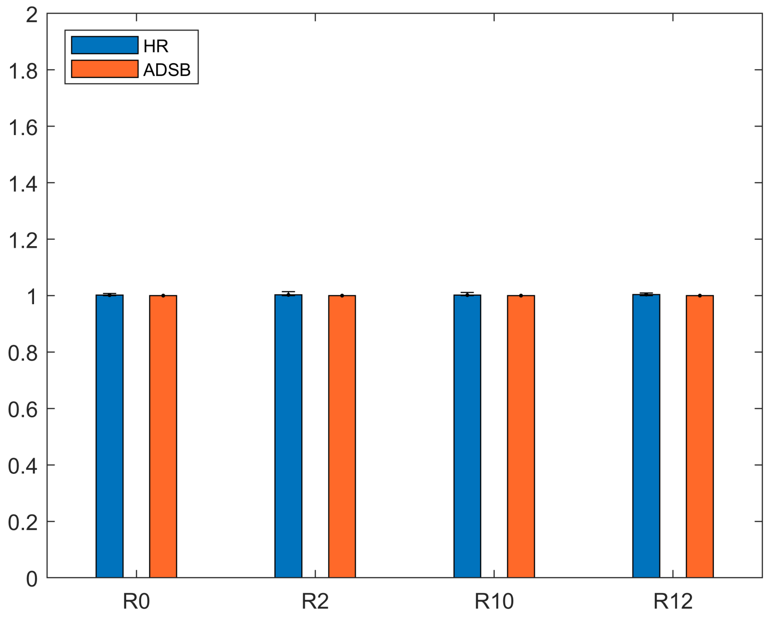

Figure S3: Comparison of the Potential Scale Reduction Factor ($psrf$) for HR and ADSB in various versions of the *E. coli* core model.

References

[1] Saa, P.A., Nielsen, L.K.: Fast-snp: a fast matrix pre-processing algorithm for efficient loopless flux optimization of metabolic models. Bioinformatics **32**(24), 3807–3814 (2016)

[2] De Martino, D., Mori, M., Parisi, V.: Uniform sampling of steady states in metabolic networks: heterogeneous scales and rounding. PLoS one **10**(4), 0122670 (2015)

[3] Saa, P.A., Nielsen, L.K.: ll-achrb: a scalable algorithm for sampling the feasible solution space of metabolic networks. Bioinformatics **32**(15), 2330–2337 (2016)

[4] Gelman, A., Rubin, D.B.: Inference from iterative simulation using multiple sequences. Statistical science **7**(4), 457–472 (1992)

[5] Gilks, W.R., Roberts, G.O., George, E.I.: Adaptive direction sampling. Journal of the Royal Statistical Society: Series D (The Statistician) **43**(1), 179–189 (1994)

[6] Roberts, G., Gilks, W.: Convergence of adaptive direction sampling. Journal of multivariate analysis **49**(2), 287–298 (1994)

[7] Smith, R.L.: Efficient Monte Carlo procedures for generating points uniformly distributed over bounded regions. Operations Research **32**(6), 1296–1308 (1984)

[8] Bélisle, C.J., Romeijn, H.E., Smith, R.L.: Hit-and-run algorithms for generating multivariate distributions. Mathematics of Operations Research **18**(2), 255–266 (1993)

[9] Neal, R.M.: Slice sampling. The annals of statistics **31**(3), 705–767 (2003)

[10] Kiatsupaibul, S., Smith, R.L., Zabinsky, Z.B.: An analysis of a variation of hit-and-run for uniform sampling from general regions. ACM Transactions on Modeling and Computer Simulation **21**(3), 1–11 (2011)

[11] Orth, J.D., Fleming, R.M.T., Palsson, B.: Reconstruction and use of microbial metabolic networks: the core escherichia coli metabolic model as an educational guide. EcoSal Plus **4**(1) (2010). doi:10.1128/ecosalplus.10.2.1. https://journals.asm.org/doi/pdf/10.1128/ecosalplus.10.2.1
